# Aggressive Adenocarcinoma of the Cervical Esophagus: Importance of a Multidisciplinary Approach

**DOI:** 10.1155/2012/826246

**Published:** 2012-12-11

**Authors:** Adedayo Fashoyin, Gregory Hartig, William R. Schelman, Mark Ritter, Rashmi Agni, Deepak V. Gopal, Patrick R. Pfau, Jennifer M. Weiss

**Affiliations:** ^1^University of Wisconsin School of Medicine and Public Health (UWSMPH), Madison, WI 53705, USA; ^2^Section of Otolaryngology, Department of Surgery, UWSMPH, P.O. Box 7375 CSC, 600 Highland Avenue, Madison, WI 53792, USA; ^3^University of Wisconsin Carbone Cancer Center, P.O. Box 5669 CSC, 600 Highland Avenue, Madison, WI 53792, USA; ^4^Department of Human Oncology, UWSMPH, P.O. Box 0600 CSC, 600 Highland Avenue, Madison, WI 53792, USA; ^5^Department of Pathology, UWSMPH, P.O. Box 3224 CSC, 600 Highland Avenue, Madison, WI 53792, USA; ^6^Division of Gastroenterology & Hepatology, Department of Medicine, UWSMPH, 4258 UW Medical Foundation Centennial Building, 1685 Highland Avenue, Madison, WI 53705-2281, USA

## Abstract

Adenocarcinoma of the cervical esophagus is a rare tumor, in comparison to adenocarcinoma of the distal esophagus which is on the rise and is often associated with Barrett's esophagus. We present a case of aggressive cervical esophageal adenocarcinoma in a 46-year-old female with no endoscopic or histopathologic evidence of Barrett's esophagus. We discuss the possible etiology of this tumor and review the treatment options, highlighting the importance of a multidisciplinary approach to the management of this rare disease.

## 1. Introduction

Esophageal adenocarcinomas typically arise in the lower third of the esophagus with a pathogenesis linked to gastroesophageal reflux disease and subsequent development of Barrett's epithelium. However, primary adenocarcinomas in the cervical esophagus are believed to result from mucosal “cardiac” glands, submucosal glands, or heterotopic gastric mucosa. Adenocarcinoma in the cervical esophagus is a rare tumor with little known about its typical prognosis or optimal therapy, including ideal surgical management and use of neoadjuvant or adjuvant chemotherapy and/or radiotherapy. 

## 2. Case Report

**Figure 1:**
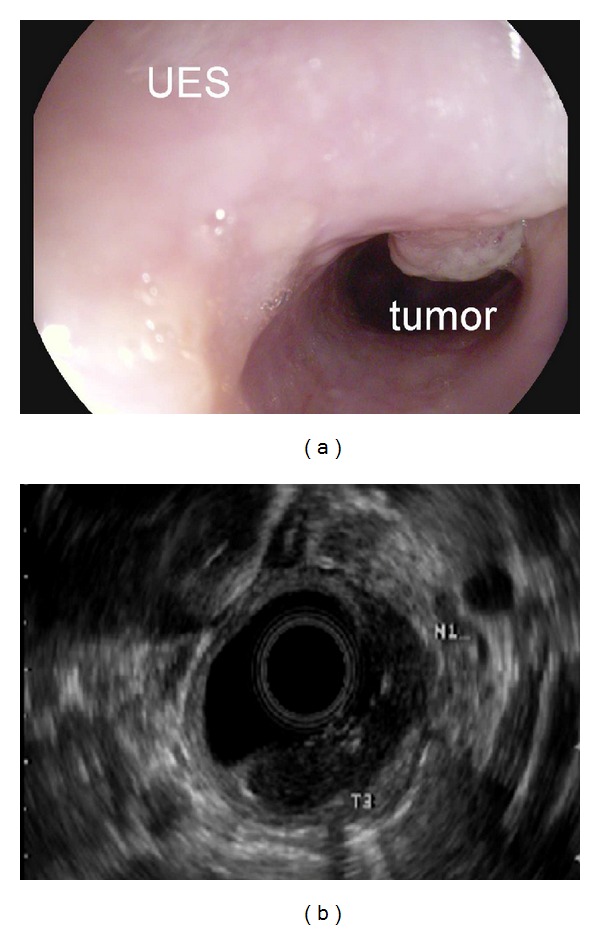
(a) Upper endoscopy highlights the close proximity of the tumor to the upper esophageal sphincter (UES). (b) Endoscopic ultrasound shows invasion of the tumor into the muscularis propria and suspicion for regional node involvement.

**Figure 2:**
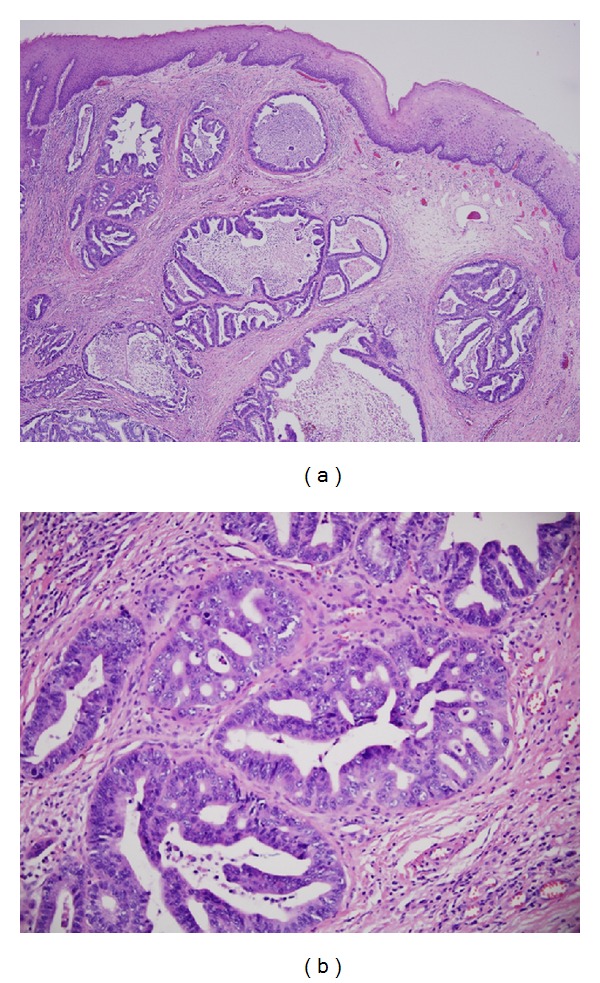
(a) H&E stain (×40 magnification) shows moderately differentiated adenocarcinoma in the deep mucosa beneath intact squamous epithelium. (b) H&E stain (×100 magnification) shows cribriform glands and micropapillae infiltrating stroma of the lamina propria with moderate cellular atypia.

A 46-year-old Caucasian woman with no significant past medical or family history presented to her primary care provider with a one-year history of sore throat and dysphagia to solid foods. She reported a remote smoking history in her teens and rare alcohol consumption. Workup included a barium swallow study with a persistent filling defect in the upper esophagus measuring 5 cm × 1.8 cm originating from the posterior right wall of the esophagus. Differential diagnosis included both benign and malignant lesions of the upper esophagus. Upper endoscopy with endoscopic ultrasound revealed an ulcerated, friable mass extending from 15 to 19 cm from the incisors with evidence of invasion into the muscularis propria and suspicion for regional node involvement (Figures 1(a) and 1(b)). Biopsies from the upper endoscopy were positive for moderately differentiated invasive adenocarcinoma (Figures 2(a) and 2(b)). 

The patient was staged as T3N1M0 after a PET/CT of the neck demonstrated a hypermetabolic esophageal mass with adjacent right paratracheal and superior mediastinal lymphadenopathy. CT scan of the chest, abdomen, and pelvis were negative for distant metastases. Six weeks of definitive/neoadjuvant chemoradiotherapy were delivered according to the Ilsen regimen (cisplatin 30 mg/m^2^ and irinotecan 65 mg/m^2^ on weeks 1, 2, 4, and 5 of radiation). Radiotherapy consisted of a total of 64.8 Gy to the primary esophageal tumor and adjacent nodes plus 39.6 Gy to superior mediastinal nodes. Follow-up PET/CT 14 weeks after completion of definitive chemoradiation therapy revealed continued hypermetabolic activity in the proximal esophagus and repeat upper endoscopy revealed residual tumor. Otolaryngology performed a cervical esophagectomy, lymph node dissection, and left radial forearm microvascular free tissue transfer reconstruction of the cervical esophagus. The pathologic specimen contained moderately differentiated adenocarcinoma of the esophagus undermining the squamous epithelium, with metastasis to two of six paratracheal lymph nodes, but no evidence of Barrett's esophagus or identifiable vestiges of heterotopic gastric mucosa. 

Five months after surgery, she developed difficulty with an anastomotic stricture that was dilated endoscopically and determined to be a result of local tumor recurrence. Nine months after the initial surgery, salvage laryngopharyngectomy with a right radial forearm flap reconstruction was performed. The patient's course continued with a second locoregional recurrence five months after salvage surgery, necessitating further chemotherapy/limited field radiotherapy (first low-dose cisplatin, then capecitabine due to an urticarial rash, and 50.4 Gy). A third recurrence five months later in a right subclavicular node was treated with resection and concurrent capecitabine/limited field radiation. Most recently, she had evidence of multifocal recurrence nearly two years after the initial chemoradiation. She is currently participating in a phase II clinical trial with an oral Aurora kinase inhibitor and has stable disease based on radiographic imaging after six treatment cycles. 

## 3. Discussion

Squamous cell carcinoma predominates in the cervical esophagus; adenocarcinoma in the cervical esophagus is extremely rare with, to our knowledge, less than 30 cases reported in the literature. Adenocarcinoma of the distal esophagus, in contrast, is common and the relative prevalence has been increasing from 1.7% to 10% historically to upwards of 50% of all malignant tumors of the esophagus in more recent studies [1, 2]. Reasons for this shift in histology are not well established, but may be due to the simultaneous rise in Barrett's esophagus (BE), which is a risk factor for distal esophageal adenocarcinoma. Adenocarcinomas of the cervical esophagus can arise from mucosal “cardiac” glands, submucosal glands, BE, and heterotopic gastric mucosa (HGM) [1, 3, 4]. HGM is thought to occur when embryologic bidirectional replacement of the esophageal columnar epithelium by squamous mucosa fails to be completed. HGM is most often asymptomatic and found incidentally during endoscopic procedures for unrelated symptoms in approximately 3.8–10% of the adult population [5, 6]. On endoscopy as well as pathologic specimen, there was no evidence of BE in our patient, so it is possible that her tumor originated from HGM. 

No standard therapy exists for primary cervical esophageal adenocarcinoma. Treatment strategies published in the few case reports range from endoscopic mucosal resection to laryngo-pharyngo-esophagectomy with lymph node dissection with or without neoadjuvant or adjuvant chemoradiotherapy. However, the number of cases reported in the current literature is too small and the details of their staging and treatment too inconsistent to postulate whether these tumors have a different prognosis than the more common distal esophageal adenocarcinomas [1]. Our patient presented with a relatively large, ultrasound-staged T3N1 tumor with a subsequent clinically aggressive course characterized by multiple locoregional recurrences despite combined modality neoadjuvant therapy with surgery. Our case highlights the likelihood that improvements in outcomes will require the best efforts of a multidisciplinary management of this rare disease with gastroenterology, otolaryngology, thoracic surgery, medical oncology, radiation oncology, and pathology.

## References

[1] Alrawi SJ, Winston J, Tan D (2005). Primary adenocarcinoma of cervical esophagus. *Journal of Experimental and Clinical Cancer Research*.

[2] Noguchi T, Takeno S, Takahashi Y, Sato T, Uchida Y, Yokoyama S (2001). Primary adenocarcinoma of the cervical esophagus arising from heterotopic gastric mucosa. *Journal of Gastroenterology*.

[3] Lauwers GY, Scott GV, Vauthey JN (1998). Adenocarcinoma of the upper esophagus arising in cervical ectopic gastric mucosa: rare evidence of malignant potential of so-called ‘inlet patch’. *Digestive Diseases and Sciences*.

[4] Abe T, Hosokawa M, Kusumi T (2004). Adenocarcinoma arising from ectopic gastric mucosa in the cervical esophagus. *American Journal of Clinical Oncology*.

[5] Poyrazoglu OK, Bahcecioglu IH, Dagli AF, Ataseven H, Celebi S, Yalniz M (2009). Heterotopic gastric mucosa (inlet patch): endoscopic prevalence, histopathological, demographical and clinical characteristics. *International Journal of Clinical Practice*.

[6] Borhan-Manesh F, Farnum JB (1991). Incidence of heterotopic gastric mucosa in the upper oesophagus. *Gut*.

